# Regions of hepatitis C virus E2 required for membrane association

**DOI:** 10.1038/s41467-023-36183-y

**Published:** 2023-01-26

**Authors:** Ashish Kumar, Tiana C. Rohe, Elizabeth J. Elrod, Abdul G. Khan, Altaira D. Dearborn, Ryan Kissinger, Arash Grakoui, Joseph Marcotrigiano

**Affiliations:** 1 Structural Virology Section, Laboratory of Infectious Diseases, National Institute of Allergy and Infectious Diseases, National Institutes of Health, Bethesda, MD 20892 USA; 2 Emory National Primate Research Center, Division of Microbiology and Immunology, Emory Vaccine Center, Emory University School of Medicine, Atlanta, GA 30329 USA; 3 Center for Advanced Biotechnology and Medicine, Rutgers, The State University of New Jersey, Piscataway, NJ 08854 USA; 4 Research Technology Branch, National Institute of Allergy and Infectious Diseases, National Institutes of Health, Hamilton, MT 59840 USA

**Keywords:** X-ray crystallography, Hepatitis C virus, Structural biology, Membrane structure and assembly

## Abstract

Hepatitis C virus (HCV) uses a hybrid entry mechanism. Current structural data suggest that upon exposure to low pH and Cluster of Differentiation 81 (CD81), the amino terminus of envelope glycoprotein E2 becomes ordered and releases an internal loop with two invariant aromatic residues into the host membrane. Here, we present the structure of an amino-terminally truncated E2 with the membrane binding loop in a bent conformation and the aromatic side chains sequestered. Comparison with three previously reported E2 structures with the same Fab indicates that this internal loop is flexible, and that local context influences the exposure of hydrophobic residues. Biochemical assays show that the amino-terminally truncated E2 lacks the baseline membrane-binding capacity of the E2 ectodomain. Thus, the amino terminal region is a critical determinant for both CD81 and membrane interaction. These results provide new insights into the HCV entry mechanism.

## Abstract (web summary)

Hepatitis C virus (HCV) uses a hybrid entry mechanism. Upon exposure to low pH, envelope glycoprotein E2 releases an internal loop into the host membrane. Here we show the amino terminal region is a critical determinant for membrane interaction, providing insights into the HCV entry mechanism.

## Introduction

Hepatitis C virus (HCV) is a leading cause of chronic hepatitis, cirrhosis, and hepatocellular carcinoma with significant morbidity and mortality rates in humans^1^. The World Health Organization estimates that there are 1.5 million new HCV infections each year and 58 million people worldwide are chronically infected^2^. In the United States, annual acute HCV infections increased by 387% from 2010 to 2019 in association with rising addiction to intravenously administered opioids^3^. The recently approved direct-acting antiviral drugs are highly effective against all genotypes of HCV^4^, but once cured, a person can become reinfected. In the context of addiction and intravenous drug use, lasting protection from HCV infection is an unmet medical need.

Cluster of differentiation 81 (CD81)^5^, scavenger receptor class B type I (SR-BI)^6^, claudin-1 (CLDN)^7^, and occludin (OCLN)^8^ are cellular factors necessary for HCV entry. Blocking HCV binding to CD81 is the primary means of antibody-mediated neutralization^9^. CD81 is ubiquitously expressed on a variety of cell lines, suggesting a role secondary to hepatocyte-specific receptor binding^10,11^. CD81 is an integral membrane protein with four transmembrane helices and two extracellular loops, denoted as the small and large extracellular loop (SEL and LEL, respectively). Low pH treatment does not inactivate HCV^12^. Incubation with both CD81-LEL and low pH creates a conformational change in HCV that irreversibly diminishes the ability of the virus to enter permissive cells^13^. Pre-treatment with either low pH buffer or CD81-LEL increases HCV infectivity, indicating that each of these conditions represents a necessary priming step^13^.

**Fig. 1:**
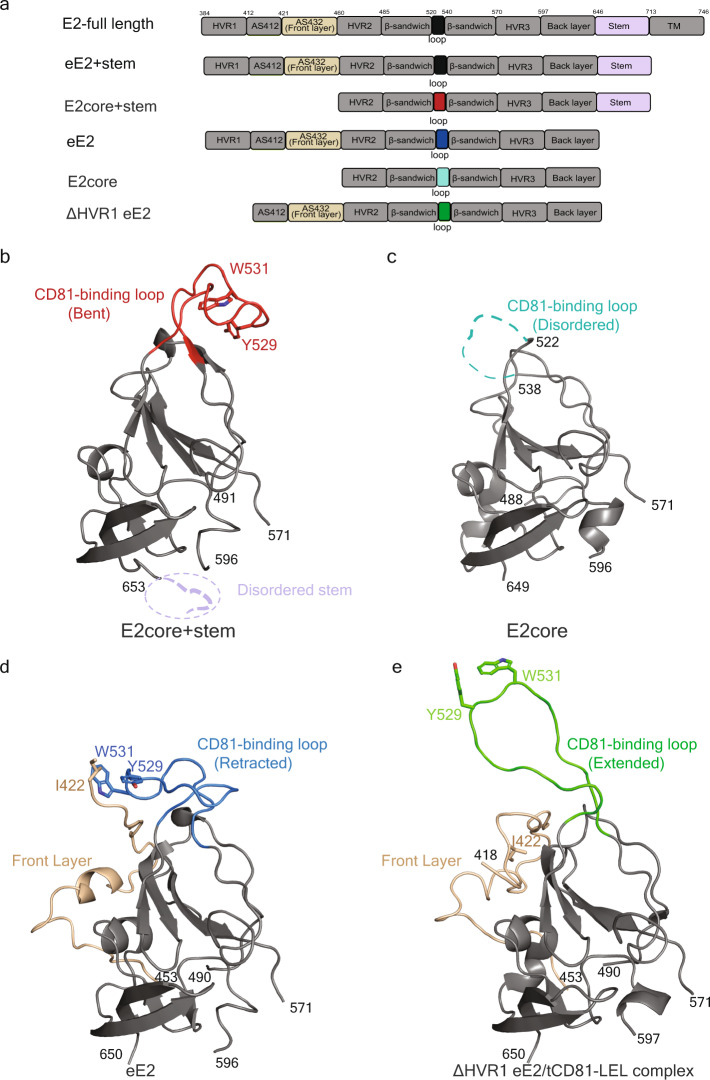
Overview of HCV E2. **a** Schematic representation of full-length E2 (isolate J6, genotype 2a), and various fragments used in structural and functional studies. **b** X-ray crystal structure of E2core+stem (PDB ID: 8DK6). The final model consists of residues 491–571 and 596–653 of E2core+stem (**c**) The E2core structure (PDB ID: 4WEB) consists of residues 488–522, 538–571, and 596–649. **d** The eE2 structure (PDB ID: 7MWW) consists of residues 422–453, 490–571, and 596–650, and **e** the ΔHVR1 eE2/tCD81-LEL (PDB ID: 7MWX) consists of residues 418–453, 490–571, and 597–650. Each structure (**b**–**e**) was determined in the presence of non-neutralizing 2A12 Fab antibody (not shown). 2A12 Fab heavy chain and light chain residues consist of residues 1–219 and 1–218, respectively. The CD81-binding loop (520–539) region is color-coded according to **a** for E2core+stem, E2core, eE2, ΔHVR1 eE2/tCD81-LEL and front layer with red, cyan, blue, green, and wheat, respectively. Tyr529, Trp531 residues at the tip of the CD81-binding loop, and Ile422 of the front layer are also highlighted. Disordered regions are represented with a dashed line. The E2 orientation in **b**–**e** is approximately the same. The residue numbering for each chain built in the final structural models is given.

While the HCV envelope glycoproteins 1 and 2 (E1 and E2) mediate receptor binding and entry, E2 interacts specifically with CD81 and SR-BI^5,6^. E2 is a type I membrane protein with an amino (N) -terminal, soluble glycoprotein and a carboxy (C) -terminal, transmembrane helix (TM) (Fig. 1a). Note that the numbering is based on the J6 sequence (genotype 2a), studied here, and varies slightly among genotypes. The central E2core is a globular, folded domain that includes a disulfide-stabilized immunoglobulin sandwich flanked by the hypervariable region 1 (HVR1), antigenic site (AS) 412, front layer, and HVR2 on the N-terminus and a stem region on the C-terminus^14^ (Fig. 1a). The overall architecture of the E2core (residues 460–646) includes a central immunoglobin β-sandwich inner sheet (residues 485-519) followed by a CD81-binding loop (residues 520–539) and a short β-sandwich sheet (residues 540–570). The E2core retains the overall E2 architecture of the ectodomain but is incapable of binding to CD81^15^.

Recently, the structures of E2 in the presence and absence of non-productive host tamarin CD81-LEL (tCD81-LEL) at low pH were reported^16^. In the absence of CD81, the ectodomain of E2 (eE2) has an internal loop (the CD81- binding loop) tucked against Ile422 of the front layer. Upon binding to CD81 at low pH, eE2 undergoes two conformational changes: the front layer interacts with CD81 by folding AS412 around CD81, and the CD81-binding loop stretches away from the core. The CD81-binding loop presents two invariant hydrophobic residues (Tyr529 and Trp531) at the tip of the loop, which are critical for interaction with the host membrane^16^.

Here we characterize the role of the front layer and the stem on CD81-LEL and membrane binding. Size exclusion chromatography and a liposome flotation assay demonstrated that the E2 front layer is a critical region for both CD81-LEL and membrane interaction. The X-ray crystal structure of the E2core+stem, lacking the HVR1, AS412, front layer, and TM regions (Fig. 1a), in the presence of a non-neutralizing antibody, 2A12, was determined. In the structure, the stem region is disordered while the CD81-binding loop is in a bent conformation, sequestering the Tyr529 and Trp531 residues within the center of the loop. The extension of the CD81-binding loop may be more nuanced than previously considered and its flexibility likely plays an important role in viral entry and fusion.

## Results

### CD81-LEL binding and structure of E2core + stem fragment

**Fig. 2:**
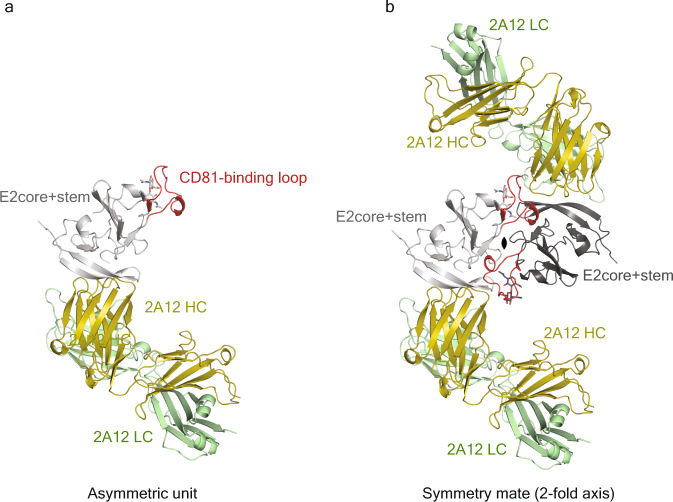
Crystal packing of the E2core+stem/2A12 Fab complex. **a** The asymmetric unit of the E2core+stem/2A12 Fab complex is shown. **b** The crystal packing of the E2core+stem/2A12 Fab complex, highlights a symmetry mate around a twofold axis of rotation into the page (black ellipse). The CD81-binding loop (red) is packed against a symmetry mate, holding the loop in the bent conformation. HC and LC referred to the heavy chain and light chain of 2A12 Fab, respectively.

All previous HCV E2 structures determined by X-ray crystallography omitted the stem (646–712) and the TM anchor (713–746) regions to increase protein solubility and stability for crystal formation^15–20^. To understand the structural role of the stem region, crystallization trials were performed on various E2 fragments in the presence and absence of the 2A12 Fab. Crystals of the E2core+stem (456–713) fragment bound to 2A12 Fab were determined to ~2.5 Å resolution (Table 1). The final model consists of residues 491–571 and 596–653 of E2core+stem as well as heavy chain residues 1–219 and light chain residues 1–218 of 2A12 Fab. Alignment of the E2core+stem structure with E2core, eE2, and the ∆HVR1 eE2/tCD81-LEL complex yielded a root-mean-square deviation (RMSD) of 0.49 Å, 0.46 Å, and 0.80 Å, respectively for similar carbon alpha positions (Fig. 1)^15,16^. The E2core+stem structure had no interpretable density for most of the stem region (residues 653–713). In the recent E1E2 structure, the stem region is in complex with E1, suggesting that the folding of this region may be E1 dependent^21^. The E2core+stem structure shows continuous electron density for the CD81-binding loop (residues 520–539) (Supplementary Fig. 2), which is distinct from previously reported structures (Fig. 1) and stabilized by crystal packing (Fig. 2).

**Table 1:** Data collection and refinement statistics for E2core + stem/2A12 Fab complex

	E2core + stem/2A12 Fab complex
Data collection*	
Space group	P22_1_2_1_
Cell dimensions	48.97, 125.54, 157.32
*a*, *b*, *c* (Å)	
(°)	90, 90, 90
Resolution (Å)	48.97–2.45(2.58–2.45)
*R*_sym_	0.109 (0.582)
*I*/σ *I*	8.8 (3.1)
Completeness (%)	97.8 (99.1)
Redundancy	4.0 (4.2)
CC (1/2)	0.991 (0.571)
Refinement	
Resolution (Å)	48.82–2.45 (2.54–2.45)
No. reflections	35532 (3508)
*R*_work_/*R*_free_	0.203/0.231
No. atoms	
Protein	575
Ligand/ion	28
Water	180
*B*-factors (Å^2^)	
Protein	54.01
Ligand/ion	91.87
Water	47.92
R.m.s. deviations	
Bond lengths (Å)	0.003
Bond angles (°)	0.69
Ramachandran plot (%)	
Favored	96.12
Allowed	3.53
Outliers	0.35

*Single crystal was used for each data collection. Values in parentheses are for highest-resolution shell.

### Large movement of the CD81-binding loop

**Fig. 3:**
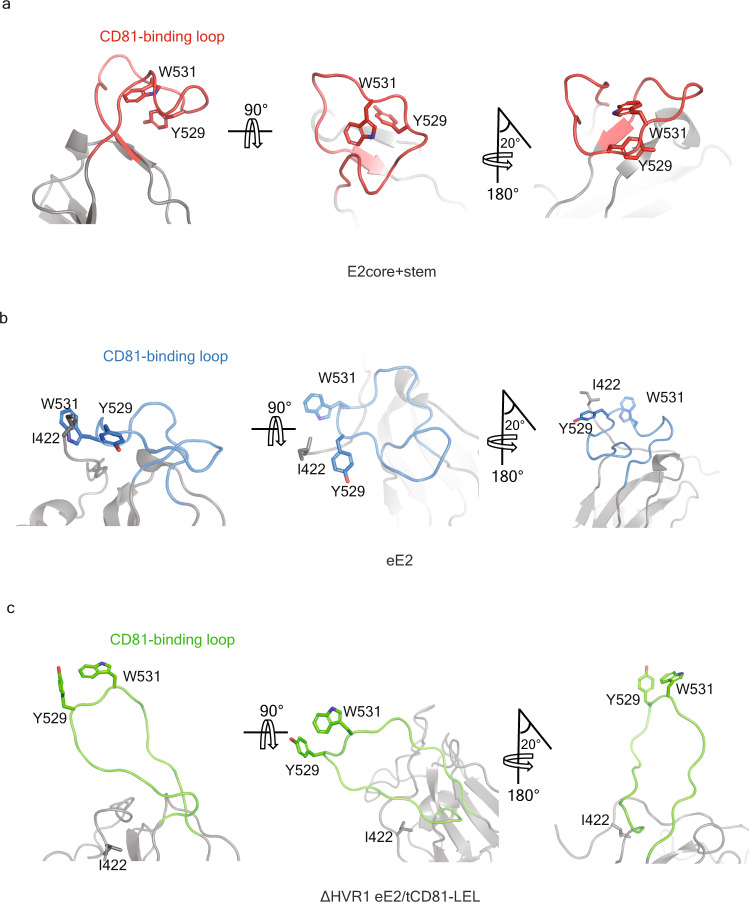
Orientation of CD81-binding loop of E2. **a** Bent (E2core+stem, PDB ID: 8DK6), **b** retracted (eE2) (PDB ID: 7MWW), and **c** extended (ΔHVR1 eE2) (PDB ID: 7MWX) in three different orientations. For clarity, Y529, W531, and I422 are highlighted and **c** bound tCD81-LEL is not shown.

The E2core+stem structure represents the fourth structure of an E2 fragment from the HCV isolate J6 bound to the crystallization chaperone 2A12 Fab^15,16^ (Fig. 1). As a result, comparing structures of different E2 lengths from the same genotype in the presence or absence of tCD81-LEL unveils important mechanistic details. The major structural difference between the four E2 structures is the orientation of the CD81-binding loop (Fig. 1b–e). Removing the CD81-binding loop from the superposition calculation lowered the RMSD to less than 0.3 Å for similar carbon alpha atoms; therefore, the presence of the front layer, HVR1, and stem has no effect on the globular fold of the E2 core region. The CD81-binding loop in the E2core+stem structure is bent towards the back layer of E2 (Figs. 1b and 3a).

**Fig. 4:**
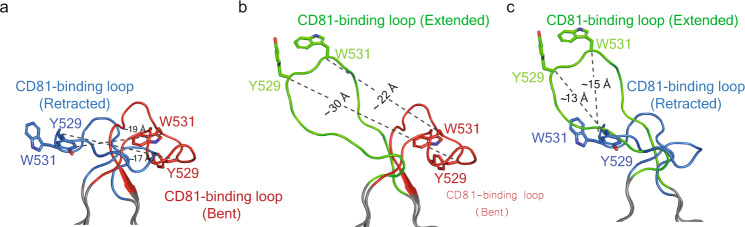
Structural comparison of the CD81-binding loop. The superposition of **a** the bent CD81-binding loop of E2core+stem (red) with the retracted CD81-binding loop of eE2 (blue), **b** the extended CD81-binding loop of ΔHVR1 eE2 (green) and bent CD81-binding loop of E2core+stem (red), and **c** the extended CD81-binding loop of ΔHVR1 eE2 (green) and retracted CD81-binding loop of eE2 (blue). **b**, **c** tCD81-LEL bound to ΔHVR1 eE2 (green) is not shown. Adjacent backbone residues are gray. The location of Y529 and W531 side chains are shown as heteroatom sticks, and the distances are provided.

To better understand the extent of the CD81-binding loop movement, the loop was compared with the corresponding region from the other E2 structures. Deletion of HVR1, AS412, and the front layer in the E2core structure (PDB ID 4WEB) resulted in the CD81-binding loop being disordered (Fig. 1c)^15^. In the eE2 structure, without CD81, the front layer and specifically residue Ile422 orients the CD81-binding loop in the retracted position (Figs. 1d and 3b). In the presence of low pH and tCD81-LEL, the AS412 region of ΔHVR1 eE2 becomes ordered and residue Ile422 moves ~9 Å, coinciding with an extended CD81-binding loop (Figs. 1e and 3c). The distance between CD81-binding loop residues, Tyr529 or Trp531, in E2core+stem (bent) and eE2, containing the front layer, in the absence of CD81-LEL (retracted) are ~17 Å and ~19 Å, respectively (Fig. 4a). Comparing the extended CD81-binding loop of ΔHVR1 eE2, from the complex with tCD81-LEL, with the bent E2core + stem demonstrated a ~30 Å and ~22 Å distance movement of Tyr529 and Trp531, respectively (Fig. 4b). In addition, the orientation of the loop is bent in the opposite direction relative to CD81. For reference, the movement of the CD81-binding loop in the absence and presence of tCD81-LEL is provided (Fig. 4c).

### CD81 binding and membrane association requires the E2 front layer

Because the CD81-binding loop was present in each of the four constructs used in structure determination but adopts distinct conformations concurrent with changes to the AS412/front layer and stem region, we hypothesized that these differences regulate CD81 binding. E2core, alone, fails to bind human CD81-LEL (hCD81-LEL) while eE2 bound to hCD81-LEL with lower affinity compared to tCD81-LEL^15^. We, therefore, probed whether the E2 stem region could affect hCD81-LEL binding. The E2core+stem was incubated with human hCD81-LEL at low pH and subsequently analyzed by size exclusion chromatography (SEC). A low pH buffer was used since the E2 affinity for hCD81-LEL is increased in the presence of low pH^16^. Addition of the stem to the E2core failed to bind hCD81-LEL in the presence or absence of 2A12 Fab (Supplementary Fig. 1). These results identify the front layer as a critical determinant for CD81-LEL interactions, consistent with the recent eE2/tCD81-LEL structure^16^.

**Fig. 5:**
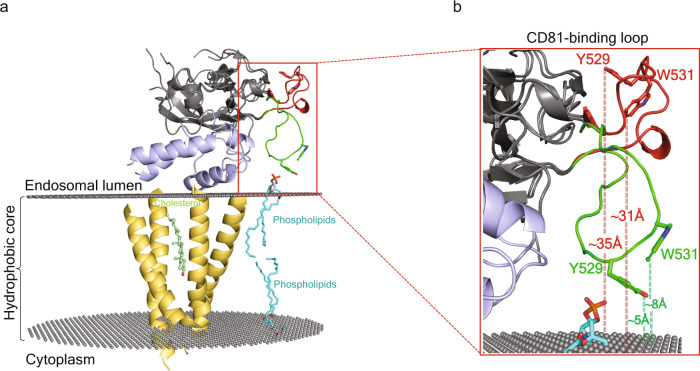
Model of E2 on a membrane-embedded, full-length CD81. **a** The full-length, human CD81 structure (ribbon diagram with the transmembrane helices colored yellow) and coordinated cholesterol molecule (light-green heteroatom sticks) (PDB ID: 5TCX) were docked into an idealized palmitoyloleoylphosphatidylcholine (POPC) membrane bilayer^44^. Parallel planes of gray spheres represent the carbonyl moieties that define the hydrophobic core of the membrane bilayer, and representative phospholipids are docked into the bilayer and shown as cyan heteroatom sticks. Ribbon diagrams of CD81-LEL (sky blue) and CD81-binding loop of extended (ΔHVR1 eE2) and bent (E2core+stem) structures are shown in green and red, respectively. **b** The panel is a magnification of the CD81-binding loop and part of the membrane showing the relative distances (dotted lines) of Tyr529 and Trp531 from the hydrophobic membrane core are labeled.

**Fig. 6:**
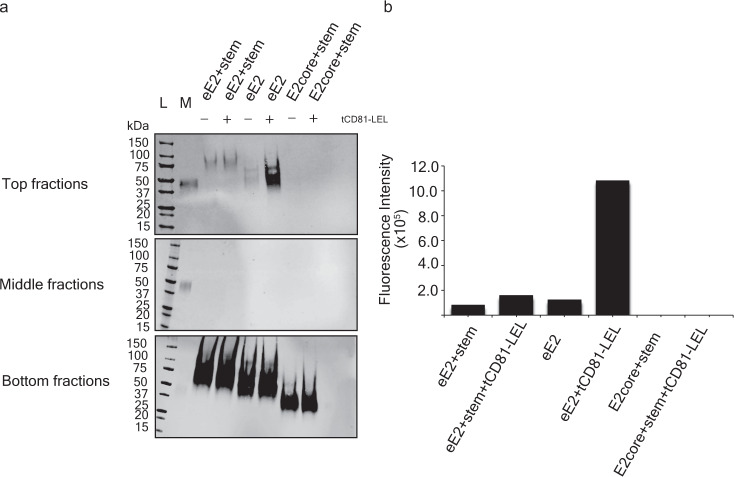
Interaction of various E2 constructs with membranes. **a** Western blots of the top, middle and bottom fractions from the liposome flotation assay. The protein molecular weight marker and eE2 loading control marker are marked as L and M, respectively. The flotation assay was performed in three independent experiments, and one assay is presented. Each experiment showed the same trend; that is, no flotation of E2core+stem with and without tCD81-LEL at low pH while a marked increase in signal in the presence of tCD81-LEL for eE2 and less enhancement with eE2+stem. For gel source data, see Supplementary Fig. 3. **b** Quantification of the top-fraction Western blot with arbitrary units.

HCV eE2 exhibits baseline membrane binding that is increased by both acidification and CD81 interaction and eliminated by mutation of Ile422, Tyr529, or Trp531^13,16^. Either of the two models could explain the interplay between the N-terminal region of E2 and the CD81-binding loop for membrane insertion (Fig. 5). If extending the CD81-binding loop is necessary and sufficient for membrane binding, then deleting the regulatory, N-terminal region will release the CD81-binding loop, resulting in an E2 protein that efficiently binds membranes in the absence of CD81. Alternatively, if the N-terminal region assists in presenting the CD81-binding loop in a manner more conducive to membrane insertion then deleting the N-terminal region will result in an E2 protein that is incapable of binding membranes. A liposome flotation assay was used to evaluate the effect of the front layer and stem of E2 on membrane binding. Briefly, eE2, eE2+stem (Fig. 1a), or E2core+stem was incubated with liposomes in the presence or absence of tCD81-LEL at pH 5.0, separated in a sucrose gradient, and detected by Western blot with an E2core-specific antibody (Fig. 6, Supplementary Fig. 3). tCD81-LEL was used in the flotation assay since tamarin CD81 supports HCV infection and binds E2 with higher affinity^16,22,23^. Free proteins remain at the bottom of the sucrose gradient, whereas liposome-bound proteins migrate to the top of the gradient. eE2 shows a dramatic liposome association at low pH in the presence of tCD81-LEL, while the addition of the stem region (eE2+stem) has a more modest increase in signal with the addition of tCD81-LEL. This could be due to the stem region (60 residues) being disordered in the absence of E1, which inhibits membrane association. Conversely, the E2core+stem failed to show membrane association and flotation at low pH regardless of the presence of tCD81-LEL. A liposome flotation performed on eE2 with or without HVR1 showed equivalent binding to liposomes at low pH in the absence and presence of tCD81-LEL. Thus HVR1 has a negligible effect on the ability of E2 to interact with membrane (Supplementary Fig. 4). The results from the liposome flotation assays (Fig. 6 and Supplementary Fig. 4) underscore the important role the front layer plays in organizing the CD81-binding loop for membrane association. The flotation of eE2 is consistent with previous findings that alanine substitution of Ile422 of the front layer (which abuts the Tyr529 in the retracted form of the CD81-binding loop) fails at membrane-association^16^. In E2core+stem structure, the CD81-binding loop is bent in the opposite direction with Tyr529 and Trp531 sequestered within the loop (Supplemental Fig. 2). In summary, the front layer of E2, CD81 receptor, and low pH are all integral requirements for properly presenting the CD81-binding loop for membrane binding.

## Discussion

Here we describe the regions of HCV E2 necessary for CD81 binding and regulation of the CD81-binding loop for membrane association, yielding unique, mechanistic insights into HCV entry. Although the CD81-binding loop of E2 is involved in membrane binding, the loop is necessary but insufficient for membrane binding. The N-terminal region that abuts the CD81-binding loop in the unbound form is also necessary for membrane binding. This N-terminal region, including the AS412 and the front layer, form the CD81-binding site, which is the primary site of antibody-mediated neutralization impacting membrane binding through Ile422. We, therefore, infer that this mechanism is finely tuned rather than just a sum of its parts.

**Fig. 7:**
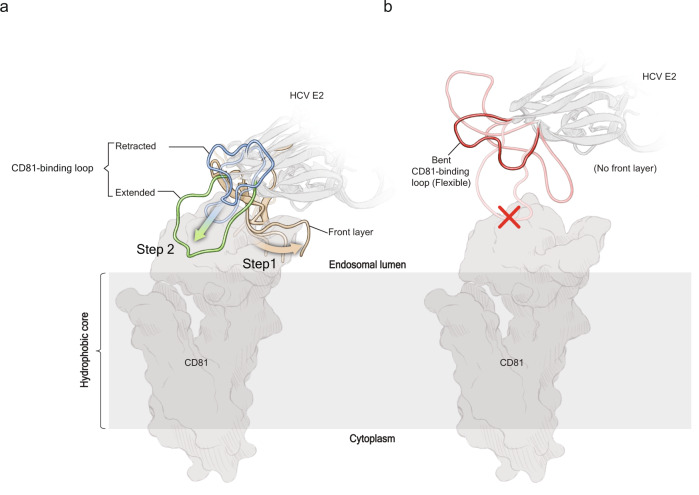
Schematic representation of HCV hybrid entry mechanism. **a** In the presence of CD81 and low pH, the front layer wraps around CD81 and then the CD81-binding loop extends out toward the host membrane. **b** Deletion of the front layer of E2 results in the release of the CD81-binding loop, which fails to bind to CD81 even at low pH, and loses the ability to interact with membranes.

Here we show the structure of the E2core+stem fragment bound to the non-neutralizing Fab 2A12 determined to 2.5 Å resolution. Structures of E2core+stem, E2core, eE2, and ΔHVR1 eE2/tCD81-LEL complex present unique mechanistic insights during the early stages of infection and yield a model for changes that occur during receptor binding and endosomal acidification (Fig. 6). The E2core region contains the folded, globular domain. The AS412 and front layer are the critical determinants for the proper positioning of the CD81-binding loop and CD81 interaction. In the absence of CD81, Ile422 of the front layer holds the CD81-binding loop in the retracted position. Both the E2core and E2core+stem failed to bind hCD81 in vitro, demonstrating the importance of the N-terminal region (384-459) in receptor binding. HCV, like avian sarcoma leukosis virus (ASLV), uses a hybrid method of membrane attachment, which requires a cellular receptor and low pH^24^. Under these conditions, E2 undergoes two conformational changes: the front layer interacts with CD81, folding the AS412 region around CD81, and an internal loop (CD81-binding loop) then stretches out away from the core (Fig. 7a). Two invariant hydrophobic residues (Tyr529 and Trp531) at the tip of the CD81-binding loop are extended towards the host and are insert into the membrane. Deleting the E2 front layer abolishes CD81 interaction and releases the CD81-binding loop (Fig. 7b). However, the E2core+stem was incapable of membrane binding, suggesting that the CD81-binding loop alone is insufficient for membrane binding and requires the front layer for proper orientation (Fig. 6). In total, these four structures provide a mechanistic picture of CD81-binding loop flexibility, the regulatory function of the N-terminal region, and detailed understanding into the viral entry process.

All envelope viruses interact and fuse with cellular membranes to deliver the viral genome into the cell. Modeling the E2/tCD81-LEL complex onto the full-length structure of CD81 (PDB ID: 5TCX^25^) Tyr529 and Trp531 are located at the tip of the CD81-binding loop that extends toward the bilayer (Fig. 5). These residues are invariant by all major genotypes and are required for CD81 binding and viral entry^26,27^. Superposition of the E2core+stem structure onto the eE2/full-length CD81 complex places the Tyr529 and Trp531 away from the outer leaflet of the membrane at distances more than 35 Å and 31 Å from an idealized phosphatidyl carbonyl layer, respectively (Fig. 5). The N-terminal region of E2 may assist in orienting the CD81-binding loop towards the membrane. The model in Fig. 5 uses an idealized bilayer. However, the flexibility of the CD81-binding loop may be advantageous during HCV infection where the curvature of the host membrane in the endosome and presence of other factors (i.e., E1, OCLN, and/or CLDN) may necessitate loop flexibility for membrane insertion.

Viral membrane fusion proteins are organized into three classes (I, II, and III) based on structural criteria with four mechanisms of fusion triggering^24^. Currently, the HCV fusion protein has not been conclusively identified, let alone classified. The HCV E1 glycoprotein is proposed to be the fusogenic subunit of the E1E2 heterodimer because it contains a putative fusion peptide^28,29^. However, the CD81-binding loop of E2 has membrane binding properties and E2 contains an IgG-like beta-sandwich fold similar to Type II fusion proteins^24,30^. Further complicating classification, E1 and E2 are required for efficient virus/host fusion, a feature not ascribed to any class. Most likely, the CD81-binding loop is embedded in the target membrane and assists in the fusion process. All fusogenic, viral proteins are trimeric, however, no evidence of an E2 trimer has been found, albeit the TM helix, or E1 may impact oligomerization. The cryoEM structure of E1E2 glycoprotein in complex with three neutralizing Fabs was recently published^21^, which contains a single copy of the E1E2 heterodimer. Similarly, inclusion of the stem did not produce trimers in the eE2+stem structure, although this might be due to the 2A12 Fab, which interacts at the carboxyl terminus of E2 protein. Further study is warranted to shed insights into the HCV fusion process.

SR-BI and CD81 have been shown to interact directly with E2 after initial attachment to the susceptible cell. SR-BI binds through the N-terminal HVR1, whereas CD81 binds to the E2 protein through a discontinuous region that includes AS412, AS432, the CD81-binding loop, and the back layer^6,16^. However, the molecular mechanism of SR-BI interaction with E2 remains obscure. CD81 with a low pH trigger may be sufficient to induce conformational changes. There is a possibility that SR-BI might be effective in inducing conformational changes in an HCV isolate-dependent way^31,32^.

Using the secondary receptor, CD81, for fusion is advantageous to the virus and consistent with previous observations^13^. While E2 and CD81-LEL formed a complex at neutral pH and may do so at the cell surface, complex stability and homogeneity increase at low pH, consistent with endosomal acidification. tCD81-LEL adopts an open conformation wherein the D-helix is unwound, while human CD81 has been observed most often in the closed conformation, intermediate and open conformations have also been described^33^. The E2/tCD81-LEL structure is only a snapshot of the complex after acidification and before any fusion-induced conformational change. As with the description of other viral fusion proteins, additional structural and mechanistic studies are needed to adequately describe the fusion mechanism of HCV.

The HVRs of E2 include a substantial proportion of HCV sequence variants. These regions are one mode of neutralizing antibody evasion. In patients, the HVR1 changes rapidly due to antibody-driven antigenic drift. Broadly neutralizing antibodies are directed against the conserved conformational epitopes of HCV E2 that compete with the CD81-binding site. The CD81-binding loop is surface exposed and is protected from antibody-mediated neutralization by conformational obfuscation. Identification of the CD81-binding loop in membrane insertion provides a target for HCV vaccine design. The relatively high conservation of the CD81-binding loop sequence among different HCV genotypes and the observation that membrane binding loops from other viruses including HIV^34^, Ebola^35–37^, inluenza^38^, and dengue^39^ are major targets for vaccines and neutralizing antibodies suggests that the CD81-binding loop of HCV may be a potential therapeutic target. The information gained from these atomic crystal structures might be useful in the development of prophylactic HCV vaccine designs.

## Methods

### Construct design, expression, and purification of E2 fragments

The HCV isolate J6 E2 fragments (E2core+stem residues 456–713, eE2+stem residues 384–713, and eE2 residue 384–656) and CD81-LEL (residues 112–202 from human and tamarin) were expressed in HEK293T GnTI(–) cells (ATCC, CRL-3022) and purified as described previously^40^. In brief, a lentiviral vector contains a CMV promoter, a prolactin signal sequence, J6 E2 fragment as stated above, and an HRV3C cleavage site followed by C-terminal protein-A and Flag tags. The stable expressing HEK293T GnTI(–) cells were produced by lentiviral transduction. Cells were then grown in an adherent cell bioreactor (Cesco Bioengineering) for long-term growth and protein production. Supernatant media was collected every 2 days and purified by IgG affinity chromatography followed by HRV3C protease in-column digestion. The protein was purified to high quality by subtractive chromatography over GST and Q columns followed by size exclusion chromatography with a Superdex200 column (Cytiva Life Sciences). The final yield of each protein was ~5–10 mg/Lof supernatant.

### Production, purification, and production of the 2A12 Fab

The protocol was adapted from a previous study^16^. In brief, the mouse 2A12 hybridoma cell line was grown in the CELLine Classic bioreactor flask (Sigma-Aldrich). 6 mL of 6 × 10^6^ cells in Iscove’s Modified Dulbecco’s medium (IMDM) with 15% low-IgG FBS, and 10 mM HEPES pH 7.5 (culture medium) were inoculated in the inner layer of the flask. The upper membrane was supplemented with 350 mL of IMDM with 1% low-IgG FBS, and 10 mM HEPES pH 7.5 (nutrient medium). Once cell confluency reached 6 × 10^8^, culture media was collected and centrifuged at 1000 rpm for 20 min. The supernatant 2A12 media was further purified through the protein G column. Before papain digestion to produce 2A12 Fab, the purified 2A12 antibody was dialyzed in 20 mM sodium phosphate pH 7.0 and 10 mM EDTA, and cysteine-HCl was added to a final concentration of 20 mM and the pH was adjusted to 7.0. Approximately 100 μL of immobilized agar bead papain (Thermo Fisher Scientific) was used for 20 mg of 2A12 antibody and 100% digestion was achieved through incubation at 22 °C overnight by gentle inversion. Digestion reaction was stopped by removing the immobilized papain by centrifugation at 4200 × *g* at 4 °C for 20 min. Purified 2A12 Fab was achieved through a two-step purification by protein A column and Protein G column. 20 mM HEPES pH 7.5, 250 mM NaCl, and 5% glycerol were used in purification while for Protein G column bound 2A12 Fab was eluted by 0.05% TFA which was immediately neutralized by 1 M Tris pH 8.0. The 2A12 Fab was desalted into 20 mM Tris pH 8.0 for further use and long-term storage.

### Crystallization of eE2core + stem/2A12 Fab Complex

E2core+stem (residues 456–713) was reconstituted with 2A12 Fab in a 1:1.2 (w/w) and hCD81-LEL was added to it in 1:1.3 molar ratio. The complex was incubated overnight at 4 °C. The complex was purified in 20 mM sodium citrate pH 4.5, 100 mM NaCl buffer by Superdex200 column (Cytiva Life Sciences) size exclusion chromatography. The major peak (Supplementary Fig. 1) was collected and concentrated through a 3 kDa MW cut-off Amicon Ultra Centrifugal Filtration Unit (Millipore) to a final concentration of 5 mg/mL. The analysis of size exclusion chromatography peaks shows that hCD81-LEL does not bind to the complex (Supplementary Fig. 1B). The E2core+stem/2A12 Fab complex was set up on small scale, 400nL, drop size using a mosquito liquid handling system (SPT Labtech) with various crystallization screens (Hampton Research). Diffraction quality crystals were grown in Hampton HR2-139 screen, condition D10; 0.2 M sodium citrate tribasic dihydrate, 20% w/v PEG 3350, pH 8.3. Crystals were directly frozen from 96-well plates using 30% ethylene glycol as a cryoprotectant, and flash-cooled in liquid nitrogen. Data were collected at 0.979 Å at the SER-CAT 22-ID beamline at the APS, Argonne National Laboratory.

### Structure determination and refinement

E2core+stem (residues 456–713)−2A12Fab crystals belong to space group P22_1_2_1_ with unit cell parameters *a*  =  48.97 Å, *b*  =  125.54 Å, and *c*  =  157.32 Å. Molecular phases were determined by PHENIX_phaser^41^ using PDB entry 7MWW as a starting model. Unambiguous placement of the Fab heavy and light chains provided the necessary phases to extend the map to cover eE2 coordinates, using iterative rounds of model building and density modification by Coot^42^. During every iterative refinement cycle, the model was evaluated for various quality parameters. The final model was built at 2.45 Å resolution. The E2core+stem model contains residues 491–571, 596–653, and 2 N-acetylglucosamines, while residues 456–490, 572–595, and 653–713 are absent due to flexibility. The 2A12 Fab model consists of residues 1–218 and 1–219 for the light and heavy chains, respectively. The model was refined to *R*_work_ 0.203 and *R*_free_ 0.231 with 96.12% of the residues in the favored, 3.53% is allowed, and 0.35% outlier regions of the Ramachandran plot calculated using the MolProbity software^43^. The overall CC1/2 (Pearson correlation coefficient) of the processed data is 0.991, and the CC1/2 in the outer shell is 0.571. The overall completeness of data is 97.8% with 99.1% in the outer shell with redundancy 4 and 4.2, respectively. The data processing and refinement statistics are given in Table 1.

### Liposome flotation assay

The liposome flotation assay was adapted with slight modifications^16^. 15 μg of a J6 E2 fragment [eE2core+stem (residues 456–713), eE2 (residues 384–656), eE2+stem (residues 384–713), or ΔHVR1 eE2 (406–656)], was mixed with 18 μg CD81-LEL (a six-fold molar excess of CD81-LEL) and volume adjusted to 60 μL with 20 mM sodium citrate pH 5.0 and 100 mM NaCl. The samples were incubated overnight at 4 °C on ice. 54 μL of 200-nm Soy PC: Cholesterol liposomes (70:30 molar ratio) from Encapsula NanoSciences (stock 10 mM or 8 μg/μL) (CPC-610) were added and incubated at 37 °C for 1 h with gentle tapping at certain intervals. After incubation, 67 μL of 3 M KCl was added to a final concentration of 1 M KCl and incubated at 37 °C for 15 min to minimize the nonspecific electrostatic association between proteins and lipids. Then 67% (w/v) sucrose in 20 mM sodium citrate pH 5.0 and 100 mM NaCl buffer was added to a final concentration of 40% in a final volume of 500 μL. The sample was mixed gently with a 1 mL pipette and then laid under a step gradient of 0.1 mL 5% and 11.4 mL of 25% (w/v) sucrose in an Open-Top Thinwall Ultra-Clear Centrifuge Tube (Beckman Coulter, 344060). Gradients were centrifuged at 281,000 × *g* for 150 min at 4 °C in an SW40 Ti swinging bucket rotor (Beckman Coulter Optima XL-100K Ultracentrifuge). After centrifugation, each gradient was fractionated, from the top to down, into 20 fractions of 600 μL. The top or middle fractions were then concentrated in 10 kDa MW cut-off Amicon Ultra-0.5 mL Centrifugal Filter Units (Millipore) at a speed of 10,000 × *g* to a final volume of 150 μL. 15 μL 10× SDS–PAGE reducing dye was added to each of these samples for Western blot analysis.

### Western blot analysis

All the fractions were diluted with 10× SDS–PAGE reducing sample buffer to a final concentration of 1× and denatured at 95 °C for 5 min. 50 μL of each sample was run along with eE2 marker and an Odyssey Protein Molecular Weight Marker (Li-Cor) (L) on 4–20% Bis-Tris precast gels (Bio-Rad). Proteins were then transferred to PVDF membranes using a Trans-Blot Turbo Transfer System (Bio-Rad). The membrane was blocked by Intercept (PBS) Blocking Buffer (Li-Cor) for 1 h at 37 °C followed by incubating the blot with a 1:500 dilution of a purified 8A6 mouse overnight at 4 °C. Primary antibody dilution was prepared in Odyssey Blocking Buffer in PBS with 0.05% Tween 20 (Sigma-Aldrich). The secondary antibody, IRDye 800CW Goat anti-Mouse IgG (Li-Cor, 926-32210), was used at a 1:10,000 dilution. The Western blot was scanned using Li-Cor Odyssey software (v.3.0).

### Production of monoclonal antibody 8A6

Monoclonal antibody 8A6 was generated in the laboratory of Dr. Arash Grakoui (IACUC protocol number YER-2002369-070816GN, Emory University School of Medicine, Atlanta, Georgia, US). BALB/c mice were immunized intraperitoneally with 50 μg eE2 in either Complete Freund’s Adjuvant (first immunization only), or incomplete Freund’s Adjuvant bi-weekly for 8 weeks. A final immunization with 50 μg of eE2 was given intravenously four days prior to the collection of splenocytes. Hybridomas were generated using a cloned HAT-sensitive mouse myeloma cell line as a fusion partner. Proliferating hybridomas were screened for their ability to bind eE2 via ELISA, at which point 8A6 was positively identified.

### Generation and purification of 8A6 Mab

Hybridoma cells were expanded in IMDM (Hyclone) containing 10% low IgG FCS (Hyclone) and gentamicin (Gibco). Supernatant was collected and clarified by centrifugation at 4000 × *g* for 10 min at 4 °C. Monoclonal antibodies were purified via Protein G affinity column chromatography and concentrated using Amicon Ultra-15 Centrifugal Filter Units (Millipore).

### Reporting summary

Further information on research design is available in the Nature Portfolio Reporting Summary linked to this article.

## Supplementary Information

Supplementary Information

Reporting Summary

## Data Availability

The data that support this study are available from the corresponding authors upon reasonable request. The coordinates and structure factors generated in this study have been deposited in the Protein Data Bank (PDB) under accession code 8DK6 (E2 core+stem/2A12 Fab). Previously published structures used in this study can be found under accession codes 4WEB (E2 core), 7MWW (eE2 structure), 7MWX (ΔHVR1 eE2/tCD81-LEL), and 5TCX (CD81 full length).
