# Supplementary material for: Regions of hepatitis C virus E2 required for membrane association
Source: Nat Commun. 2023 Jan 26;14:433. doi: 10.1038/s41467-023-36183-y (PMC9879980; doi:10.1038/s41467-023-36183-y)
Supplement: Supplementary file 1 — Supplementary Information [file 41467_2023_36183_MOESM1_ESM.pdf]

Supplementary Information

# REGIONS OF HEPATITIS C VIRUS E2 REQUIRED FOR MEMBRANE ASSOCIATION

Ashish Kumar[1*], Tiana C. Rohe[1*], Elizabeth J. Elrod[2], Abdul G. Khan[3], Altaira D. Dearborn[1], Ryan Kissinger[4], Arash Grakoui[2,], Joseph Marcotrigiano[1]

[1] *Structural Virology Section, Laboratory of Infectious Diseases, National Institute of Allergy and Infectious Diseases, National Institutes of Health, Bethesda, MD 20892*

[2] *Emory National Primate Research Center, Division of Microbiology and Immunology, Emory Vaccine Center, Emory University School of Medicine, Atlanta, GA 30322, Atlanta, GA 30329*

[3] *Center for Advanced Biotechnology and Medicine, Rutgers, The State University of New Jersey, Piscataway, NJ 08854.*

[4] *Research Technology Branch, National Institute of Allergy and Infectious Diseases, National Institutes of Health, Hamilton, MT 59840*

* Equal contributors

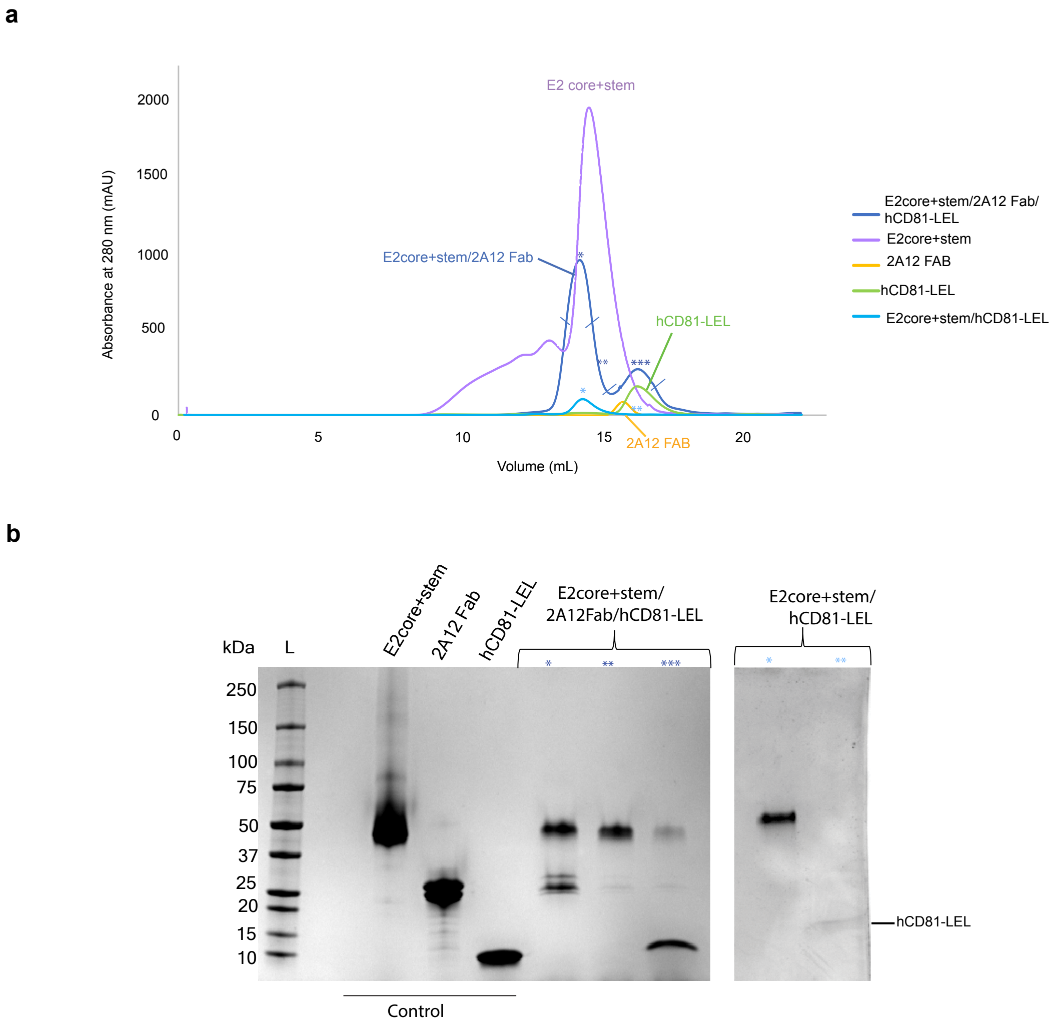


**Supplementary figure 1.** Characterization of complex formation. **a** Super-positioned size-exclusion chromatograms of E2core+stem, 2A12Fab, and hCD81-LEL (blue) or E2core+stem and hCD81-LEL (light blue) co-incubated at low pH and controls E2core+stem (violet), 2A12 Fab (orange), hCD81-LEL (green). The UV absorbance is plotted as a function of elution volume. **b** Corresponding major peaks of **a** panel (marked with asterisks) were analyzed by SDS-PAGE (left) E2core+stem/2A12 Fab/hCD81-LEL complex and (right) E2core+stem/hCD81-LEL complex). Each sample and protein molecular weight ladder (L) (Bio-rad) is labeled at the top of the well and apparent molecular weights are labeled on the left. The experiments were repeated more than three times, yielding similar results.

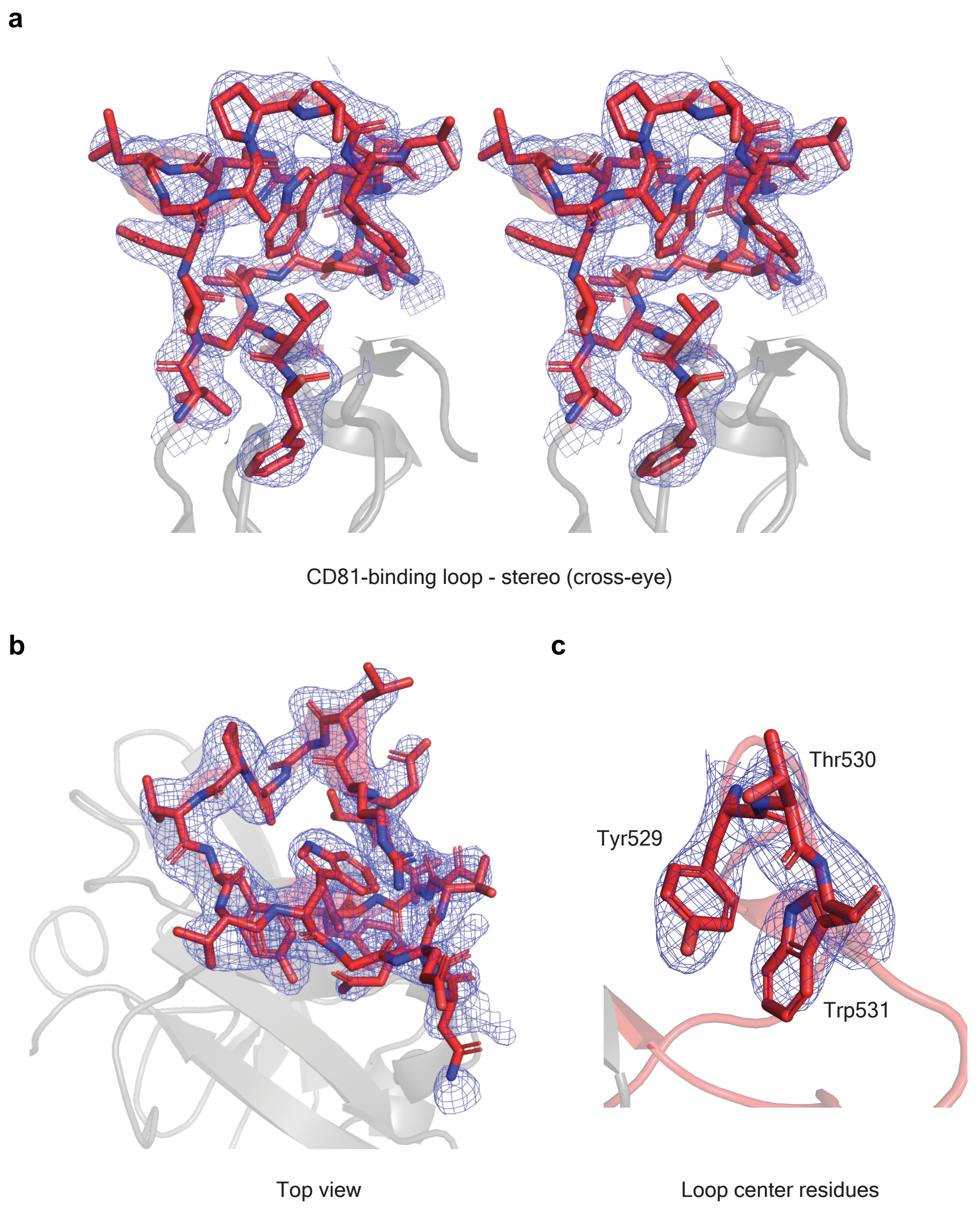


**Supplementary figure 2.** Electron density map of the CD81-binding loop from the E2core+stem structure. **a** Stereo view of the CD81-binding loop of E2core+stem structure is shown. **b** Figure shows the CD81-binding loop of E2core+stem rotated ~90° along a horizontal axis compared to **a**. **c** Electron density for the Tyr529 and Trp531 residues. The composite SA omit electron density is shown at 1 sigma.

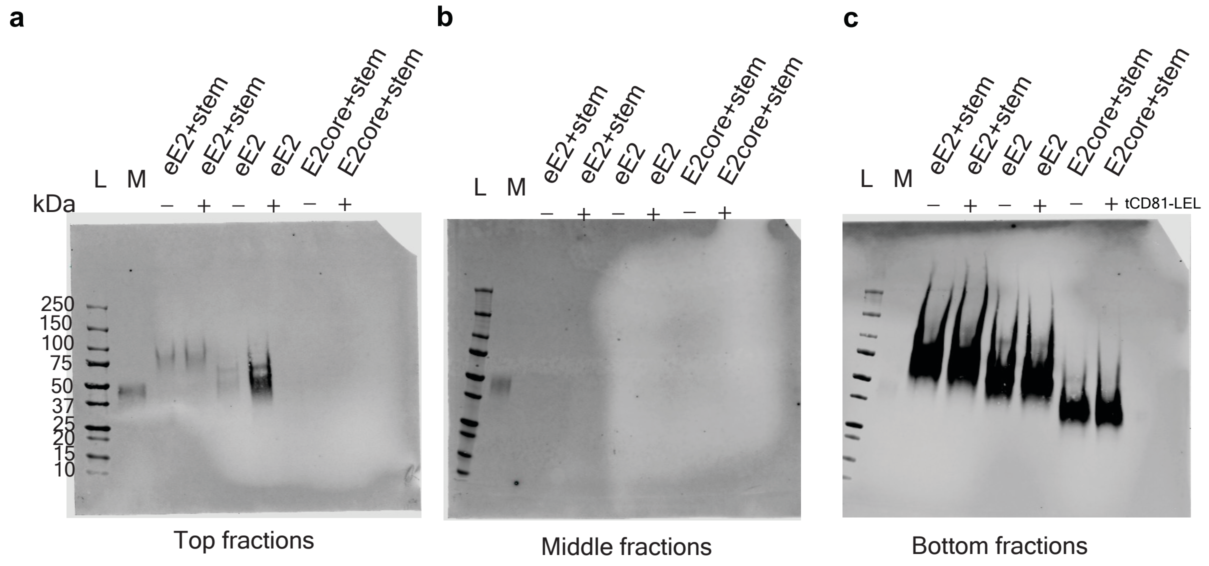


**Supplementary figure 3.** Gel source data for figure 6. **a** Anti-E2core Western blots of top, **b** middle, and **c** bottom sucrose-gradient fractions indicating liposome-binding activity via flotation. Each sample, a protein molecular weight ladder (L) (Bio-rad), and eE2 size marker (M) is labeled at the top of the well. Apparent molecular weights (left) and pre-incubation with (+) or without (-) tCD81-LEL (top) are labeled.

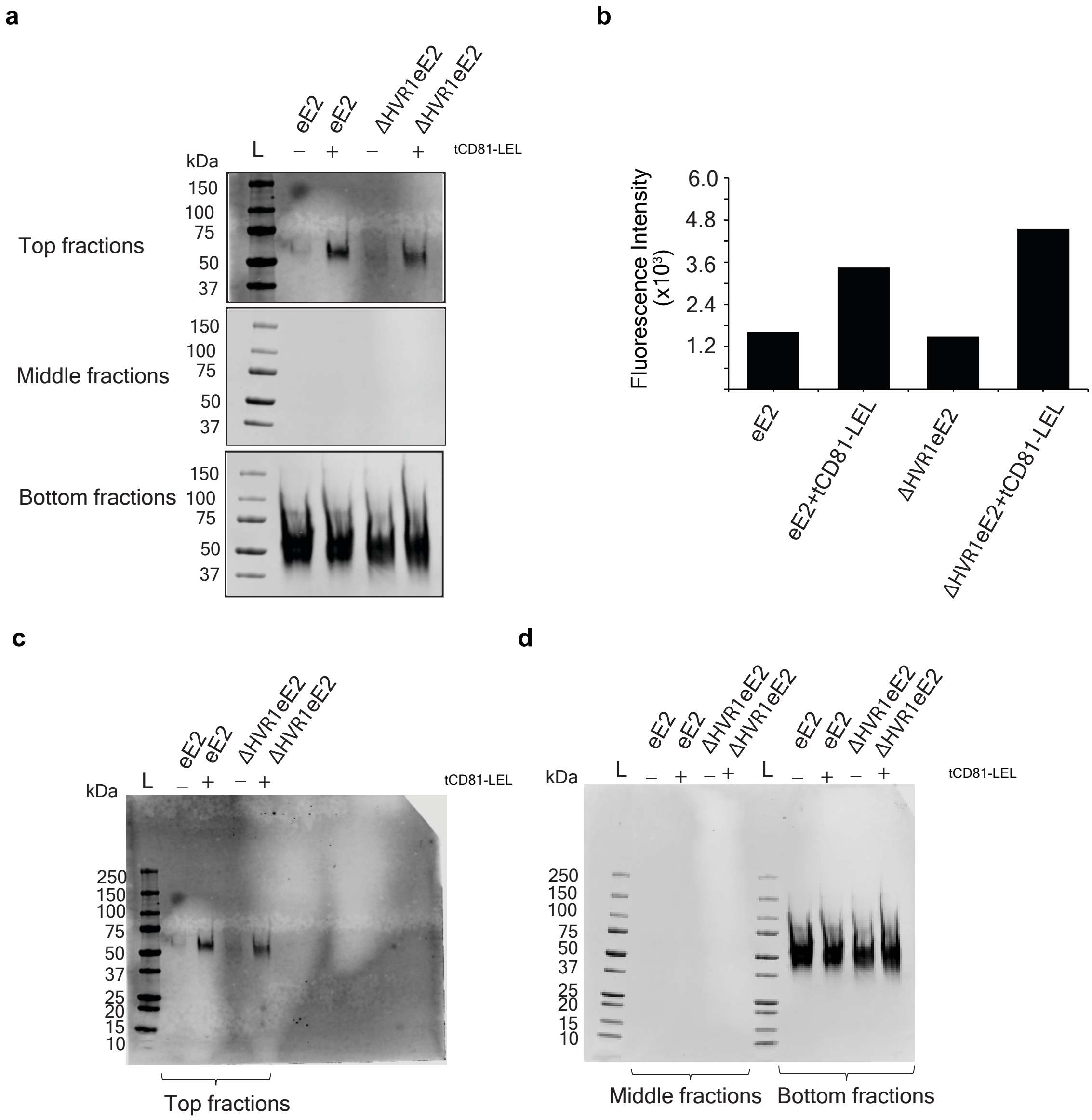


**Supplementary figure 4.** Interaction of eE2 and ΔHVR1 eE2 with the membrane at low pH with and without tCD81-LEL. **a** Cropped, and **c-d** uncropped Western blots of the top, middle, and bottom sucrose gradient fractions indicating liposome-binding activity via flotation. Each sample and a protein molecular weight ladder (L) (Bio-rad) is labeled at the top of the well. Apparent molecular weights (left) and pre-incubation with (+) or without (–) tCD81-LEL (top) are labeled. The flotation assay was performed in three independent experiments, and one assay is presented. **b** Quantification of the top-fraction Western blot with arbitrary units.
