# Supplementary material for: Regions of hepatitis C virus E2 required for membrane association
Source: Nat Commun. 2023 Jan 26;14:433. doi: 10.1038/s41467-023-36183-y (PMC9879980; doi:10.1038/s41467-023-36183-y)
Supplement: Supplementary file 2 — Reporting Summary [file 41467_2023_36183_MOESM2_ESM.pdf]

nature portfolio

Corresponding author(s): Joseph Marcotrigiano

Last updated by author(s): Jan 6, 2023

Reporting Summary

Nature Portfolio wishes to improve the reproducibility of the work that we publish. This form provides structure for consistency and transparency in reporting. For further information on Nature Portfolio policies, see our Editorial Policies and the Editorial Policy Checklist.

Statistics

For all statistical analyses, confirm that the following items are present in the figure legend, table legend, main text, or Methods section.

n/a	Confirmed	
☐	☒	The exact sample size (*n*) for each experimental group/condition, given as a discrete number and unit of measurement
☐	☒	A statement on whether measurements were taken from distinct samples or whether the same sample was measured repeatedly
☒	☐	The statistical test(s) used AND whether they are one- or two-sided<br>*Only common tests should be described solely by name; describe more complex techniques in the Methods section.*
☒	☐	A description of all covariates tested
☒	☐	A description of any assumptions or corrections, such as tests of normality and adjustment for multiple comparisons
☒	☐	A full description of the statistical parameters including central tendency (e.g. means) or other basic estimates (e.g. regression coefficient) AND variation (e.g. standard deviation) or associated estimates of uncertainty (e.g. confidence intervals)
☒	☐	For null hypothesis testing, the test statistic (e.g. *F*, *t*, *r*) with confidence intervals, effect sizes, degrees of freedom and *P* value noted<br>*Give P values as exact values whenever suitable.*
☒	☐	For Bayesian analysis, information on the choice of priors and Markov chain Monte Carlo settings
☒	☐	For hierarchical and complex designs, identification of the appropriate level for tests and full reporting of outcomes
☒	☐	Estimates of effect sizes (e.g. Cohen's *d*, Pearson's *r*), indicating how they were calculated

Our web collection on statistics for biologists contains articles on many of the points above.

Software and code

Policy information about availability of computer code

Data collection	The X-ray crystallographic data were collected at Southeast Regional Collaborative Access Team (SER-CAT) 22-ID using software developed for this beamline at the Advanced Photon Source (APS).
Data analysis	The X-ray crystallographic data were analyzed using publicly available software: Coot 0.8.92 (https://www2.mrc-lmb.cam.ac.uk), CCP4 7.0.77 (www.ccp4.ac.uk), and Phenix 1.14-3260 (http://www.phenixonline.org).

For manuscripts utilizing custom algorithms or software that are central to the research but not yet described in published literature, software must be made available to editors and reviewers. We strongly encourage code deposition in a community repository (e.g. GitHub). See the Nature Portfolio guidelines for submitting code & software for further information.

Data

Policy information about availability of data

All manuscripts must include a data availability statement. This statement should provide the following information, where applicable:

- Accession codes, unique identifiers, or web links for publicly available datasets
- A description of any restrictions on data availability
- For clinical datasets or third party data, please ensure that the statement adheres to our policy

The coordinates and structure factors for E2core+stem/2A12 Fab have been deposited into the Protein Data Bank under accession numbers 8DK6. The previous

published structures used in this study are E2core+stem (PDB ID: 8DK6), E2core structure (PDB ID: 4WEB), eE2 structure (PDB ID: 7MWW), ΔHVR1 eE2/tCD81-LEL (PDB ID: 7MWX), and the full-length structure of CD81 (PDB ID: 5TCX).

Human research participants

Policy information about studies involving human research participants and Sex and Gender in Research.

Reporting on sex and gender	N/A
Population characteristics	N/A
Recruitment	N/A
Ethics oversight	N/A

Note that full information on the approval of the study protocol must also be provided in the manuscript.

Field-specific reporting

Please select the one below that is the best fit for your research. If you are not sure, read the appropriate sections before making your selection.

☒ Life sciences ☐ Behavioural & social sciences ☐ Ecological, evolutionary & environmental sciences

For a reference copy of the document with all sections, see nature.com/documents/nr-reporting-summary-flat.pdf

Life sciences study design

All studies must disclose on these points even when the disclosure is negative.

Sample size	A single crystal was used for all the structure data. The E2core+stem/2A12 crystallographic data sets are 99.1% complete with 4.2 fold redundancy. Full details are in Extended Data Table 1.
Data exclusions	No data was excluded.
Replication	The structure was determined multiple times from different crystals. The manuscript reports the highest resolution dataset. The membrane flotation experiments were done multiple times and the results from two independent experiments are provided. The membrane flotation assays were done at least three times. All attempts were successful.
Randomization	For structure refinement, a random subset of 5% reflections was set aside by the Phenix program for calculating the Rfree value that was used to evaluate the quality of the final model. This process is not controlled and does not affect the final model calculation. No randomization was performed in other experiments.
Blinding	Blinding is not relevant to this study as knowledge of the protein sequence is needed in order to accurately determine its structure.

Reporting for specific materials, systems and methods

We require information from authors about some types of materials, experimental systems and methods used in many studies. Here, indicate whether each material, system or method listed is relevant to your study. If you are not sure if a list item applies to your research, read the appropriate section before selecting a response.

Materials & experimental systems

n/a	Involved in the study	
☐	☒	Antibodies
☐	☒	Eukaryotic cell lines
☒	☐	Palaeontology and archaeology
☒	☐	Animals and other organisms
☒	☐	Clinical data
☒	☐	Dual use research of concern

Methods

n/a	Involved in the study	
☒	☐	ChIP-seq
☒	☐	Flow cytometry
☒	☐	MRI-based neuroimaging

Antibodies

Antibodies used	2A12 and 8A6 are anti HCV E2 from genotype J6 and were generated in Dr. Arash Grakoui's lab Emory University School of Medicine

Antibodies used	against purified, recombinant protein. Secondary antibody IRDye 800CW Goat anti-Mouse IgG was purchased from a commercial source (cat. no. 926-32210 from Li-Cor).
Validation	8A6 was shown to bind E2 by ELISA and Western blot (this manuscript). 2A12 was shown to bind E2 by ELISA, isothermal calorimetry, size exclusion chromatography, and X-ray crystallography (Khan et al. Nature, 2014; Ashish et al. Nature 2021).

Eukaryotic cell lines

Policy information about cell lines and Sex and Gender in Research

Cell line source(s)	HEK293T GNTI- was purchased from ATCC (cat. no. CRL-3022) and aliquot was provided by D. Comoletti
Authentication	The cells line were not authenticated.
Mycoplasma contamination	The cells were not tested for mycoplasma contamination.
Commonly misidentified lines (See ICLAC register)	no commonly misidentified cell lines were used.
